# Prognostic role of serum thymidine kinase 1 kinetics during neoadjuvant chemotherapy for early breast cancer

**DOI:** 10.1016/j.esmoop.2021.100076

**Published:** 2021-03-10

**Authors:** A. Matikas, K. Wang, E. Lagoudaki, B. Acs, I. Zerdes, J. Hartman, E. Azavedo, J. Bjöhle, L. Carlsson, Z. Einbeigi, I. Hedenfalk, M. Hellström, T. Lekberg, N. Loman, A. Saracco, A. von Wachenfeldt, S. Rotstein, M. Bergqvist, J. Bergh, T. Hatschek, T. Foukakis

**Affiliations:** 1 Department of Oncology-Pathology, Karolinska Institutet, Stockholm, Sweden; 2 Breast Center, Theme Cancer, Karolinska University Hospital, Stockholm, Sweden; 3 Pathology Department, University Hospital of Heraklion, Heraklion, Greece; 4 Department of Clinical Pathology and Cytology, Karolinska University Laboratory, Stockholm, Sweden; 5 Department of Molecular Medicine and Surgery, Karolinska Institutet, Stockholm, Sweden; 6 Department of Oncology, Sundsvall General Hospital, Sundsvall, Sweden; 7 Department of Medicine and Department of Oncology, Southern Älvsborg Hospital, Borås, Sweden; 8 Institute of Clinical Sciences, Sahlgrenska Academy, Sahlgrenska University Hospital, Gothenburg, Sweden; 9 Division of Oncology, Department of Clinical Sciences Lund, Lund University, Lund, Sweden; 10 Department of Hematology, Oncology and Radiation Physics Skåne University Hospital, Lund, Sweden; 11 Breast Center, Södersjukhuset, Stockholm, Sweden; 12 Department of Clinical Science and Education, Karolinska Institutet, Stockholm, Sweden; 13 Biovica International, Uppsala Science Park, Uppsala, Sweden

**Keywords:** breast cancer, longitudinal, neoadjuvant chemotherapy, prognosis, thymidine kinase 1, TK1 kinetics

## Abstract

**Background:**

Emerging data support the use of thymidine kinase 1 (TK1) activity as a prognostic marker and for monitoring of response in breast cancer (BC). The long-term prognostic value of TK1 kinetics during neoadjuvant chemotherapy is unclear, which this study aimed to elucidate.

**Methods:**

Material from patients enrolled to the single-arm prospective PROMIX trial of neoadjuvant epirubicin, docetaxel and bevacizumab for early BC was used. Ki67 in baseline biopsies was assessed both centrally and by automated digital imaging analysis. TK1 activity was measured from blood samples obtained at baseline and following two cycles of chemotherapy. The associations of TK1 and its kinetics as well as Ki67 with event-free survival and overall survival (OS) were evaluated using multivariable Cox regression models.

**Results:**

Central Ki67 counting had excellent correlation with the results of digital image analysis (*r* = 0.814), but not with the diagnostic samples (*r* = 0.234), while it was independently prognostic for worse OS [adjusted hazard ratio (HR_adj_) = 2.72, 95% confidence interval (CI) 1.19-6.21, *P* = 0.02]. Greater increase in TK1 activity after two cycles of chemotherapy resulted in improved event-free survival (HR_adj_ = 0.50, 95% CI 0.26-0.97, *P* = 0.04) and OS (HR_adj_ = 0.46, 95% CI 0.95, *P* = 0.04). There was significant interaction between the prognostic value of TK1 kinetics and Ki67 (p_interaction_ 0.04).

**Conclusion:**

Serial measurement of serum TK1 activity during neoadjuvant chemotherapy provides long-term prognostic information in BC patients. The ease of obtaining serial samples for TK1 assessment motivates further evaluation in larger studies.

## Highlights

• This is a correlative analysis of a prospective phase II study on neoadjuvant chemotherapy for breast cancer.
• Serial measurement of serum TK1 activity during treatment provides independent long-term prognostic information.
• We demonstrate the validity and clinical utility of both central and automated image analysis-based Ki67 assessment.
• Finally, we explore the biologic correlations between TK1 and Ki67.

## Introduction

Following the demonstration that further adjuvant therapy improves survival in case of residual invasive breast cancer (BC) after neoadjuvant chemotherapy (NACT),^1^^,^^2^ the latter is increasingly becoming the standard of care for the treatment of early BC.^3^ As a result, optimization of NACT through the early identification of poor responders and disease progression is a priority. To this end, clinical response,^4^^,^^5^ positron emission tomography,^6^^,^^7^ tumor infiltrating lymphocytes^8^ and immune function genes^9^ have been explored as predictors of both short- and long-term outcomes. Another widely used marker of proliferation, Ki67, has also been evaluated in a number of studies which have demonstrated its pretherapy predictive^10^ and prognostic value,^11^ as well as the prognostic significance of low Ki67 expression in the post-NACT surgical specimen.^12^ The prognostic implications of Ki67 kinetics after short-term exposure to neoadjuvant endocrine therapy have also been explored and available data show that an early drop in Ki67 expression after 2 weeks of treatment predicts short- and long-term outcomes.^13^^,^^14^ However, concerns for the analytical validity and reproducibility of Ki67 assessment,^15^ as well as potential bias due to non-random missingness when evaluating on-treatment samples, have hindered its routine integration in clinical guidelines.^16^ As an effort to overcome some of these concerns, digital image analysis platforms have been developed and validated.^17^, ^18^, ^19^

Another promising biomarker is thymidine kinase (TK1), an enzyme critical for DNA synthesis and, essentially, a marker of proliferation.^20^^,^^21^ Studies have shown that patients with BC have higher levels compared with healthy controls^22^ and that higher serum TK1 activity is associated with poor BC outcomes.^23^, ^24^, ^25^ Intriguingly, TK1 kinetics during treatment have also been shown to be prognostic for outcomes in patients with metastatic BC treated with endocrine therapy,^20^^,^^26^ and for response to NACT.^27^ Serum TK1 activity exhibits several advantages as a biomarker, such as analytical and clinical validity,^20^^,^^24^^,^^28^^,^^29^ as well as ease to obtain serial samples compared with Ki67, which is a tissue-based marker.

We have previously demonstrated the feasibility and clinical validity of longitudinally assessing biomarkers during ongoing therapy.^9^ Despite its promise, no studies have evaluated TK1 kinetics during NACT as a predictor of long-term patient survival in patients with BC. The aim of this study was to investigate the association of dynamic changes of this marker following short-term exposure to chemotherapy with patient survival, as well as to further validate digital image analysis of Ki67 in an effort to circumvent the known analytical difficulties of Ki67 assessment.

## Methods

### Clinical trial, endpoints and sample collection

PROMIX was an academic, non-randomized, single arm, multicenter phase II study which was conducted in five centers in Sweden between September 2008 and November 2011 (ClinicalTrials.gov identifier NCT00957125). Its design has been previously described in detail.^9^^,^^30^ In brief, patients with human epidermal growth factor receptor 2-negative BC and tumors larger than 20 mm were enrolled in the study and were treated with neoadjuvant epirubicin 75 mg/m^2^ in combination with docetaxel 75 mg/m^2^, both administered every 3 weeks, for six cycles. Bevacizumab 15 mg/kg every 3 weeks was added to cycles 3-6 in case of non-complete clinical response. The trial including the correlative analyses was approved by the Ethics Committee at Karolinska Institutet (2007/1529-31/2), which had jurisdiction for all participating centers and by the Swedish Medical Product Agency. All patients provided written informed consent before enrollment to the clinical trial. The study was carried out in accordance with the Declaration of Helsinki.

The primary endpoints of the study were the characterization of early functional and biological changes signaling pathologic complete response (pCR), and the radiological response rate as assessed by mammography and ultrasound. Within the scope of this correlative analysis, two time-to-event endpoints were used: event-free survival (EFS), defined as the time from enrollment to study until local, regional or distant BC relapse, new contralateral BC, other malignancy or to death due to any cause, whichever occurred first; and overall survival (OS), defined as the time from enrollment to study until death due to any cause. Censoring time for those events was defined as time from enrollment until last contact (through November 2020).

Core biopsies were obtained at baseline before treatment initiation and after two cycles of NACT. Serum samples for measurement of TK1 activity were obtained immediately before and 48 h following every cycle, for the first four cycles (eight timepoints in total). Within the scope of this analysis, data gathered from baseline and 48 h after cycle 2 were used in order to correspond to the timepoints of the biopsies.

This study is reported in accordance with REporting recommendations for tumor MARker (REMARK) prognostic studies guidelines.^31^

### Ki67 staining, scanning, central reading and interpretation

Whole-tissue sections (thickness: 4 μm) were prepared from formalin-fixed paraffin-embedded tissue blocks from all patient samples and placed on positively charged Superfrost Plus microscope slides (Thermo Scientific, Waltham, MA). For the immunohistochemistry (IHC), slides were deparaffinized using xylene for 15 min and hydrated in a series of graded ethanol to water. Antigen retrieval was then carried out using Diva (BioCare Medical, Pacheco, CA) pretreatment reagent in a decloaking chamber (BioCare Medical) under pressure at 95°C for 45 min and cooled down in wash buffer. Peroxidase block, protein block, immunostaining with the rabbit primary monoclonal antibody targeting Ki67 (clone SP6, 1 : 100, BioCare Medical), primary antibody binding detection with an HRP-coupled polymer combined with 3,3′-diaminobenzidine chromogen (MACH 1 Universal HRP-Polymer Detection Kit, BioCare Medical) and counterstaining with hematoxylin were carried out in the IntelliPath FLX automated slide stainer (BioCare Medical) per manufacturer's protocol. Slides were then dehydrated in water to increasing ethanol concentration and mounted using Pertex (Histolab, Gothenburg, Sweden). Colon and skin tissues were used as positive controls and liver and kidney tissues as negative controls.

Ki67 tumor expression was evaluated centrally by an experienced certified pathologist (EL) who was blinded to the patient characteristics, Ki67 values from the diagnostic samples and outcome. Evaluation was carried out according to the recommendations provided by the International Ki67 Breast Cancer Working Group.^32^ Specifically, Ki67 score was calculated by dividing the total number of positively stained invasive tumor cell nuclei with the total number of tumor cell nuclei counted across each whole core-cut ×100. As adequate for scoring were considered core-cuts that comprised a minimum of 500 invasive tumor cells. Only nuclear staining (plus mitotic figures which are stained by Ki67) was incorporated into the Ki67 score. A nucleus was considered positively stained if presenting any definite (brown) staining above the surrounding background in the cytoplasm and extracellular matrix, regardless of the intensity of staining, and as negative when presenting only the blue counterstaining. Mitotic figures, normal ducts and lymphocytes served as the internal positive control. Foci of carcinoma *in situ*, areas of necrosis as well as non-tumoral tissue (stroma, inflammatory background) were excluded from counting.

### Digital image analysis of Ki67

The NanoZoomer-XR platform (Hamamatsu Photonics, Hamamatsu, Japan) was used at ×20 to digitize the slides with a pixel size of 0.4537 μm × 0.4537 μm. The QuPath (open source software^33^) platform was used to build an automated Ki67 scoring algorithm in BC. As the date of Ki67 staining varied within the cohort, we refined stain estimates for each digitized slide (estimate stain vectors command in QuPath). We used watershed cell detection^34^ to segment the cells in the image with the following settings: detection image: optical density sum; requested pixel size: 0.5 μm; background radius: 8 μm; median filter radius: 0 μm; sigma: 1.5 μm; minimum cell area: 10 μm^2^; maximum cell area: 400 μm^2^; threshold: 0.1; maximum background intensity: 2. In order to classify detected cells into tumor cells, immune cells, stromal cells and others (false detections, background), we used a neural network^35^ as a machine learning method with 8 hidden layers (maximum iterations: 100). The features used in the classification are described in Supplementary Table S1, available at https://doi.org/10.1016/j.esmoop.2021.100076. In order to help the algorithm to carry out an accurate classification, we also added smoothed object features at 25 μm and 50 μm radius to supplement the existing measurements of individual cells. Quality control of the algorithm to classify detected cells was carried out by a pathologist. The analysis was run on the entire tumor area on the core biopsy slide defined by a pathologist.

### Measurement of TK1 activity

The DiviTum® ELISA-based assay (Biovica, Uppsala, Sweden) that was used to measure TK1 activity has been previously described in detail.^20^^,^^24^ In short, each sample is diluted in a dilution buffer and then incubated with a reaction mixture on the assay microtiter plate. Bromodeoxyuridine (BrdU), a thymidine analogue, is phosphorylated to BrdU-monophosphate by the TK present in the sample, then further phosphorylated and incorporated in a DNA strand bound to the bottom of the wells. BrdU incorporation is detected by the ELISA technique using an anti-BrdU monoclonal antibody conjugated to alkaline phosphatase and a chromogenic substrate, producing a yellow reaction product. Absorbance is measured at 405 nm with the reference wavelength of 630 nm after 30 and 60 min of incubation. The measured optical density is proportional to the enzymatic TK1 activity of each sample, expressed as DiviTum® units per liter (Du/l), calculated from a standard curve based on calibrators of known TK activity. All samples were analyzed at Biovica laboratories in Uppsala, Sweden with no access to, nor any knowledge of, patient identity and tumor characteristics.

### Gene expression profiling and analysis

RNA was extracted from core biopsies obtained at baseline and cycle 2, as well as from the surgical specimen, and profiled on the Illumina Human HT-12 v4.0 Expression BeadChip (Illumina Inc., San Diego, CA), as described previously.^30^ The association between serum TK1 and tumor gene expression patterns was analyzed. Baseline gene expression data (GEO:GSE87455) were collapsed to gene level using a nonspecific filter keeping only the probesets with the highest interquartile range in the case of multiple mappings to the same Entrez Gene ID. The association between log10-transformed serum TK1 and tumor gene expression was assessed with gene-wise linear multivariable models including IHC subtype as an adjustment variable and moderated *t*-tests using the Bioconductor limma package. Enrichment analyses of the reactome gene set collection in the Molecular Signatures Database (Broad Institute, Cambridge, MA) were carried out using the gene set enrichment analysis (GSEA) software (Broad Institute) with genes pre-ranked according to the moderated *t*-test statistics. Multiple testing was controlled by calculating the expected false discovery rate according to Benjamini & Hochberg. Corresponding analysis for baseline tumor Ki67 was carried out with shifted log10-transformed Ki67 [log10(Ki67 + 1)] as a response variable.

### Statistical analysis

The association between serum TK1 and clinical-pathological characteristics at baseline was analyzed with a general multivariable linear regression model with log10-transformed TK1 as the response variable and shifted log10-transformed tumor Ki67 values [log10(Ki67 + 1)], tumor size (continuous variable), lymph node involvement (no, yes) and IHC subtype (luminal-like and triple-negative BC) as explanatory variables.

In this exploratory correlative study, associations between candidate biomarkers and EFS or OS were analyzed. Given that DiviTum® did not provide a formal cut-off value, log10-transformed serum TK1 at baseline and its kinetics were regarded as a binary variable according to median value. Cox proportional risk modeling was fitted to estimate crude and multivariable-adjusted hazard ratios (HRs) and 95% confidence intervals (CIs), and the HRs for ‘high’ versus ‘low’ groups were calculated. Variables were included in the multivariate proportional hazard regression full model if *P* values <0.1 (two-sided) in univariate analyses, which included age (continuous), tumor size (<50 mm and ≥50 mm), lymph node involvement (negative and positive), estrogen receptor (ER) status (negative and positive) and central Ki67 by median value (<12% and ≥12%). Final multivariable models were determined from the full model by applying backward model selection. ER status violated the proportional hazards assumption that was examined using the Schoenfeld residual test,^36^ and as such, the stratification Cox regression models were employed. Potential effect modification by important variables such as Ki67 and IHC subtype was examined by adding the cross-product of each effect modifier with TK1 kinetics in the multivariable-adjusted model. Likelihood ratio tests were conducted, and *P* values <0.1 were considered as an indicator of significant effect modification.

*P* values reported (two-sided) <0.05 were considered statistically significant. All analyses were conducted using R software (version 4.0.1).

## Results

### Patient characteristics and outcomes

In total, 150 patients were enrolled to the PROMIX trial. One patient died due to toxic death after the first chemotherapy cycle and was excluded from further analyses.^37^ The patient characteristics of the study cohort have been previously described.^30^ The distribution of patient characteristics according to baseline TK1 activity is presented in Table 1, while data availability is presented in Supplementary Figure S1, available at https://doi.org/10.1016/j.esmoop.2021.100076. The median follow-up for the entire cohort (*n* = 150) was 80 months (interquartile range, 64-110 months), while the median follow-up for patients with available baseline TK1 data (*n* = 125) was 79 months (interquartile range, 64-110 months).

**Table 1:** Distribution of clinicopathologic characteristics of patients enrolled in the PROMIX trial, according to baseline TK1 activity (median value as cut-off)

	TK1 at baseline		*P* value
	Low	High	
	*N* = 63 (100%)	*N* = 62 (100%)	
Mean age (SD), years	48.54 (8.29)	49.74 (11.30)	0.50^a^
Tumor size, mm			
<50	20 (31.7)	17 (27.4)	0.8^b^
≥50	41 (65.1)	42 (67.7)	
Unknown	2 (3.2)	3 (4.8)	
Lymph node status			
Negative	54 (85.7)	51 (82.3)	0.78^b^
Positive	9 (14.3)	11 (17.7)	
Grade			
I	2 (3.2)	1 (1.6)	0.48^c^
II	22 (34.9)	16 (25.8)	
III	12 (19.0)	18 (29.0)	
Unknown	27 (42.9)	27 (43.5)	
ER status			
Negative	14 (22.2)	22 (35.5)	0.15^b^
Positive	49 (77.8)	40 (64.5)	
PR status			
Negative	28 (44.4)	28 (45.2)	0.99^b^
Positive	35 (55.6)	34 (54.8)	
Ki67, %^d^			
<12	37 (58.7)	24 (38.7)	0.04^b^
≥12	26 (41.3)	38 (61.3)	
pCR			
No	55 (87.3)	52 (83.9)	0.77^b^
Yes	8 (12.7)	10 (16.1)	

ER, estrogen receptor; pCR, pathological complete response; PR, progesterone receptor; SD, standard deviation; TK1, thymidine synthetase 1.

a Student's *t*-test.

b Chi-square test.

c Fisher's exact test.

d Ki67 was categorized by its median value, where some missing values were imputed by microarray MKI67 expression data. Multiple imputation of missing Ki67 values (*n* = 36) was carried out by a univariate logistic regression model that included MKI67 expression value (continuous) from the baseline microarray RNA data, and 10 cycles were repeated to produce a final dataset.

### Clinicopathologic and biologic correlations of TK1 and Ki67

At baseline, 106 patients had available data for Ki67 with both central and automated counting. Central Ki67 counting had an excellent correlation with the results of the digital image analysis (*r* = 0.814). However, both were poorly correlated with Ki67 from the diagnostic samples by local assessment (*r* = 0.234 for the central counting and *r* = 0.214 for the digital image analysis). Baseline TK1 activity was modestly correlated with central Ki67 (*r* = 0.315, analysis of variance *P* = 0.02), but not with tumor size (*P* = 0.379), lymph node status (*P* = 0.355) or IHC subtype (*P* = 0.144).

**Figure 1:**
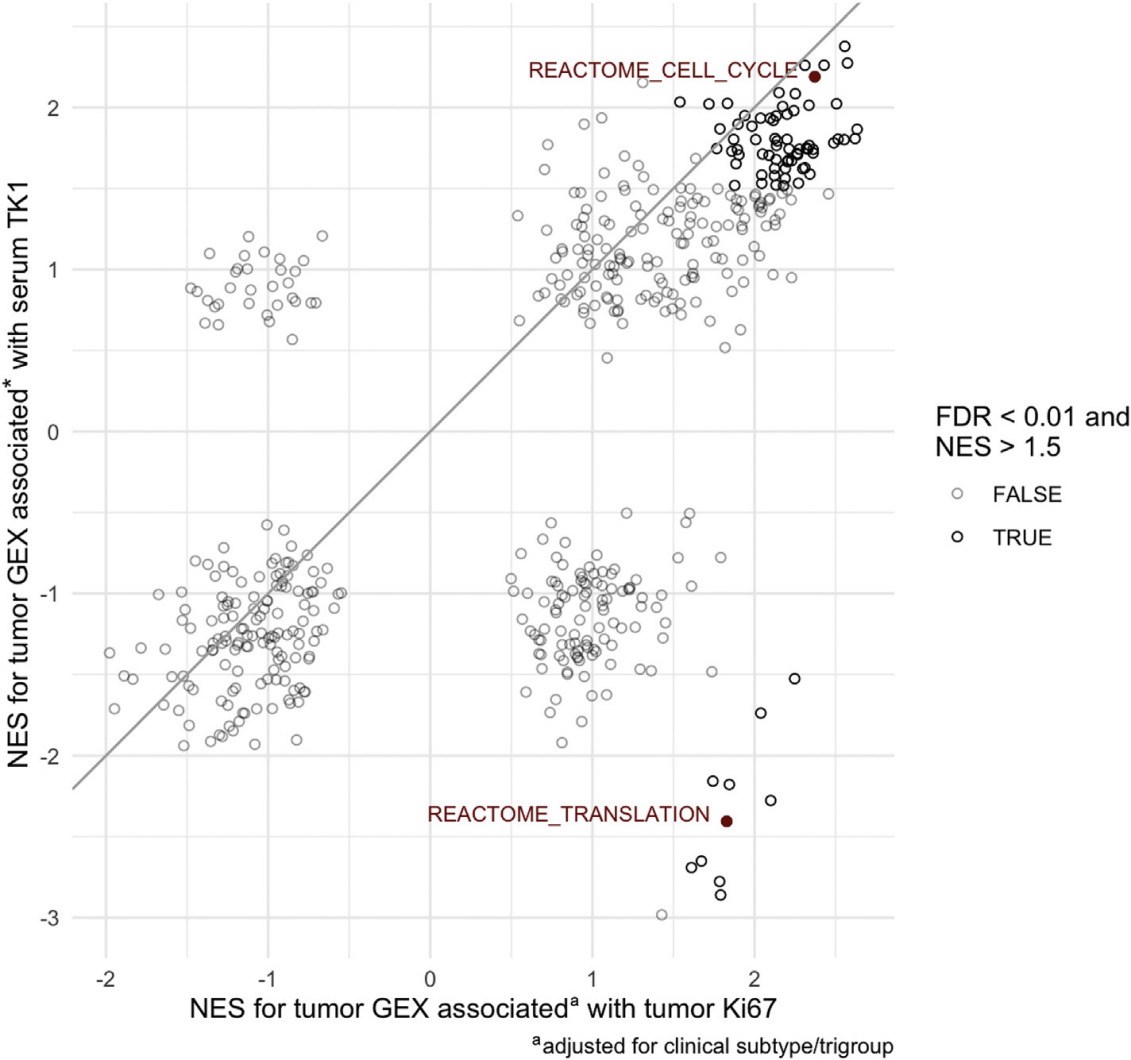
Gene set enrichment analysis of baseline tumor gene expression associated with serum TK1 and tumor Ki67, respectively. FDR, false discovery rate; GEX, gene expression; NES, normalized enrichment score; TK1, thymidine kinase 1.

In order to further investigate the biologic correlates of TK1 activity, we carried out an exploratory GSEA. Both Ki67 and TK1 were associated with similar gene expression patterns. In particular, gene sets related to cell cycle progression were enriched in tumors with higher Ki67 expression and TK1 activity (Figure 1). An exception of differential expression patterns between the two markers concerned translation-related gene expression, which was positively correlated with Ki67 and negatively with TK1. A full list of highly concordant and discordant gene sets is provided in Supplementary Table S2, available at https://doi.org/10.1016/j.esmoop.2021.100076.

### Baseline TK1 and Ki67 as long-term prognosticators

In univariate analysis, higher baseline TK1 activity was prognostic for worse EFS (HR = 1.89, 95% CI 1.04-3.42, *P* = 0.04) and OS (HR = 1.97, 95% CI 1.05-3.70, *P* = 0.04). However, when adjusted for clinicopathologic variables, baseline TK1 activity was no longer statistically significantly associated with long-term patient outcomes, as shown in Tables 2 and 3. In contrast, among the patients who also had available data on baseline central Ki67 assessment, Ki67 ≥ 12% (more than the median value) was independently prognostic for worse OS [adjusted HR (HR_adj_) = 2.72, 95% CI 1.19-6.21, *P* = 0.02] when adjusted for tumor size, nodal status, age, baseline TK1 and stratified for ER status.

**Table 2:** Univariate and multivariable analyses for TK1 activity (baseline and change to post-cycle 2) with event-free survival as clinical endpoint

TK1^a^	No. of cases	5-year EFS (months) (95% CI)	Cox proportional hazards regression			
			Unadjusted model		Adjusted model^b^	
			HR (95% CI)	*P*	HR (95% CI)	*P*
TK1 at baseline^a^						
Low	63	76.0 (66.1-87.4)	1.00 (reference)		1.00 (reference)	
High	62	58.1 (47.0-71.7)	1.89 (1.04-3.42)	0.04	1.56 (0.83-2.92)	0.16
TK1 increase from baseline to cycle 2^a^						
Low	56	53.6 (42.0-68.4)	1.00 (reference)		1.00 (reference)	
High	55	77.8 (67.4-89.8)	0.38 (0.20-0.72)	0.003	0.50 (0.26-0.97)	0.04

CI, confidence interval; EFS, event-free survival; HR, hazard ratio; TK1, thymidine synthetase 1.

a Low/high delineations of TK/TK kinetics were determined according to their median value.

b Stratified by estrogen receptor status (negative, positive) due to proportional hazards assumption violation (*P* = 0.04) and adjusted for tumor size (≥50 mm, <50 mm), regional lymph node (negative, positive).

**Table 3:** Univariate and multivariable analyses for TK1 activity (baseline and change to post-cycle 2) with overall survival as clinical endpoint

TK1^a^	No. of cases	5-year OS (months) (95% CI)	Cox proportional hazards regression			
			Unadjusted model		Adjusted model^b^	
			HR (95% CI)	*P*	HR (95% CI)	*P*
TK1 at baseline^a^						
Low	63	85.6 (77.3-94.7)	1.00 (reference)		1.00 (reference)	
High	62	74.2 (64.1-85.9)	1.97 (1.05-3.70)	0.04	1.55 (0.80-2.98)	0.19
TK1 increase from baseline to cycle 2^a^						
Low	56	71.4 (60.5-84.3)	1.00 (reference)		1.00 (reference)	
High	55	85.3 (76.4-95.2)	0.36 (0.18-0.72)	0.004	0.46 (0.22-0.95)	0.04

CI, confidence interval; HR, hazard ratio; OS, overall survival; TK1, thymidine synthetase 1.

a Low/high delineations of TK/TK kinetics were determined according to their median value.

b Stratified by estrogen receptor status (negative, positive) due to proportional hazards assumption violation (*P* = 0.04) and adjusted for tumor size (≥50 mm, <50 mm), regional lymph node (negative, positive).

### TK1 kinetics following two cycles of NACT and its prognostic value

**Figure 2:**
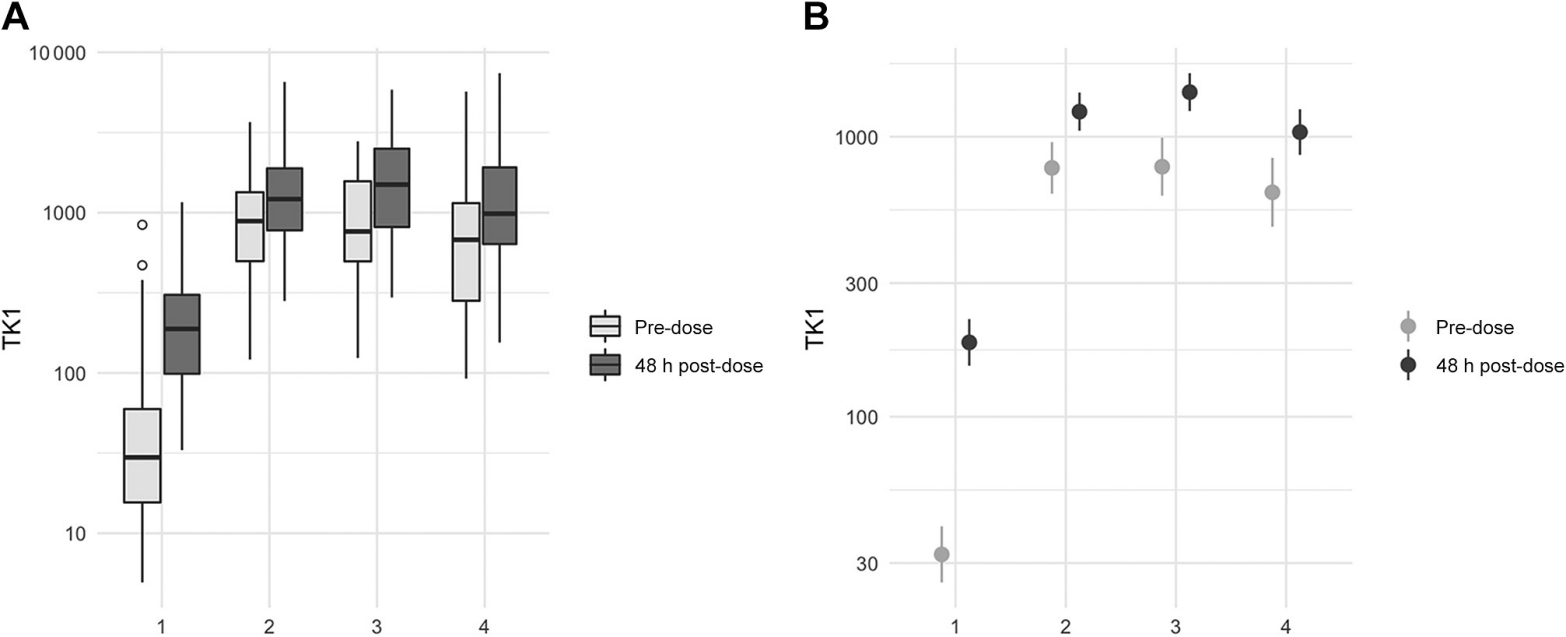
(A) Boxplot of TK1 over time and (B) mean and 95% confidence interval of log10-transformed TK1 at each timepoint. TK1, thymidine kinase 1.

Figure 2 depicts TK1 activity over time. In general, it was greater at the after dose compared with the before dose measurements and after an initial increase from cycle 1 to cycle 2, TK1 activity remained relatively stable over time. In multivariable analysis, increase in TK1 activity following two cycles of chemotherapy compared with baseline was associated with improved patient survival (Tables 2 and 3 and Supplementary Figure S2, available at https://doi.org/10.1016/j.esmoop.2021.100076). Patients with a larger increase had improved EFS (HR_adj_ = 0.50, 95% CI 0.26-0.97, *P* = 0.04) and OS (HR_adj_ = 0.46, 95% CI 0.95, *P* = 0.04) compared with patients with a lower increase in TK1 activity. Sensitivity analyses using Ki67, age and pCR as additional variables confirmed these results (Supplementary Tables S3 and S4, available at https://doi.org/10.1016/j.esmoop.2021.100076). Additional exploration using per 10-fold increase of TK1 activity as a metric is shown in Supplementary Table S5, available at https://doi.org/10.1016/j.esmoop.2021.100076.

### Effect of BC subtype and baseline Ki67 on the prognostic value of TK1 increase from baseline to cycle 2

The prognostic information provided by the increase of TK1 activity during NACT was similar across IHC subtypes (p_interaction_ 0.95). However, significant interaction was observed between increase in TK1 and baseline Ki67 (p_interaction_ 0.04). Patients with baseline Ki67 exceeding the median value (12%) had a significantly improved EFS per higher increase in TK1 activity (HR_adj_ = 0.34, 95% CI 0.12-0.96), but not those with Ki67 < 12% (HR_adj_ = 1.44, 95% CI 0.44-4.75), when adjusted for tumor size, nodal status and stratified for ER status.

## Discussion

In this correlative analysis of a prospective non-randomized trial, we aimed to evaluate the prognostic value of the longitudinal assessment of TK1 activity as measured in serial blood samples. This study is the continuation of another correlative analysis using material from the same phase II trial where we demonstrated that the longitudinal assessment of immune function provides useful predictive information, and we emphasized the role of the immune microenvironment as a driver of chemosensitivity.^9^ In the present study, dynamic changes in TK1 activity during NACT were found to predict long-term outcomes independently of other known prognosticators, including pCR.

The ease of obtaining serial measurements and thus longitudinally assessing TK1 makes it an attractive potential early marker of response. In contrast, tissue-based biomarkers are characterized by the need to obtain new biopsies, analytical difficulties such as non-representative material or poor cellularity, and interobserver variability, although standardization improves yield and their performance.^38^ Literature on TK1 activity suggests that it may have diverse interpretations: proliferation, monitoring during treatment, response to therapy or even resistance to cyclin-dependent kinase 4/6 inhibitors,^39^, ^40^, ^41^ depending on the clinical setting in which it is assessed. No available data, however, answer the question whether changes in proliferation during NACT can exclusively explain the prognostic value of TK1 and its kinetics. As a result, we aimed to simultaneously evaluate another well-established marker of proliferation, Ki67, and their clinical and biologic correlations. Of note, longitudinally assessing Ki67 during NACT was not possible in this study due to high rates of possibly informative missingness and thus, risk for bias, since tumors that respond well to chemotherapy are less likely to have evaluable Ki67.

Our results shed some light on the interplay between proliferation as evaluated by Ki67 expression and TK1 activity. The two markers were positively associated with gene sets related to cell cycle progression, indicating that proliferation explains—at least partly—TK1 activity. This is further supported by the observed effect that Ki67 expression had on the prognostic value of TK1 kinetics. However, the dynamic behavior of TK1 under the effect of cytotoxic chemotherapy differed compared with that of Ki67 according to the available literature.^12^ Since we demonstrated that Ki67 and TK1 are both correlated with cell cycle progression, the association of greater increase in TK1 activity with better prognosis seems counterintuitive and even more so when considering prior studies showing that a decrease in TK1 activity correlated with tumor response to endocrine therapy.^20^^,^^26^^,^^41^ Nevertheless, the plausibility of this finding—that TK1 kinetics behave differently during cytotoxic chemotherapy—is bolstered by similar data in non-small-cell lung cancer showing better prognosis when an increase in TK1 activity was observed following chemotherapy,^42^ while this early spike in TK1 activity has also been demonstrated in metastatic BC as well.^43^ Whether this effect signifies its release from cancer cells due to chemotherapy,^44^ the role of TK1 as a metabolic marker of exposure of cancer cells to chemotherapy or of leukocyte recovery is unclear.^42^^,^^45^ Intriguingly, *in vitro* data have shown that a rise in TK1 is observed when BC cells are treated with doxorubicin,^46^ further supporting its potential role as a marker of response independently of the effects of chemotherapy on proliferation.

Although several lines of evidence support the role of Ki67 as a prognostic and predictive marker in BC,^10^, ^11^, ^12^, ^13^, ^14^ even as a marker for selection of candidates for adjuvant chemotherapy,^47^ both its widespread implementation and recommendation by guidelines have been plagued by well-described analytical weaknesses and interobserver variability in scoring.^32^ In this study we underscore the significance of central Ki67 counting, which was poorly correlated with the local assessment of the marker's expression, but was found to be highly correlated with the independent automated analysis. Based on these findings and in agreement with previously published literature,^48^^,^^49^ central Ki67 assessment should be considered as the optimal option in clinical and translational studies exploring biomarkers in BC. At the same time, digital image analysis based on this and other studies^50^ could potentially become the new standard, greatly improving turnover times while simultaneously providing robust results and circumventing the known shortcomings of Ki67 assessment.

This study is, to our knowledge, the only study that longitudinally assessed TK1 as a putative long-term prognosticator in BC, both at baseline and following short-term exposure to NACT. Additional strengths are the prospective nature of the study and long-term follow-up, a prerequisite in BC biomarker studies due to the long natural history of the disease. Nevertheless, limitations that should be acknowledged include the exploratory nature of the study and the lack of further validation of this retrospective analysis of prospectively collected data. In addition, the relatively small number of patients and survival events, as well as the limited number of available paired measurements, may have masked associations with outcomes or led to chance findings. Moreover, although we speculate on the underlying biology of TK1 kinetics, further studies should be undertaken in order to confirm our speculations. Finally, although our results describe the prognostic value of TK1 kinetics during conventional, anthracycline- and taxane-containing NACT, patients included in PROMIX were treated with additional bevacizumab between cycles 3 and 6, which is not approved for the indication, thus further underscoring the exploratory nature of our findings.

In conclusion, serial measurement of TK1 activity measured in patients' blood provides prognostic information in BC patients. The ease of obtaining serial samples for TK1 assessment motivates further evaluation in larger studies.
