# Supplementary material for: Prognostic role of serum thymidine kinase 1 kinetics during neoadjuvant chemotherapy for early breast cancer
Source: ESMO Open. 2021 Mar 10;6(2):100076. doi: 10.1016/j.esmoop.2021.100076 (PMC7957142; doi:10.1016/j.esmoop.2021.100076)
Supplement: Supplementary Material [file mmc1.docx]

**Supplementary Material to**

**Prognostic role of serum thymidine kinase 1 kinetics during neoadjuvant chemotherapy for early breast cancer**

Alexios Matikas, Kang Wang, Eleni Lagoudaki, Balazs Acs, Ioannis Zerdes, Johan Hartman, Edward Azavedo, Judith Bjöhle, Lena Carlsson, Zakaria Einbeigi, Ingrid Hedenfalk, Mats Hellström, Tobias Lekberg, Niklas Loman, Ariel Saracco, Anna von Wachenfeldt, Samuel Rotstein, Mattias Bergqvist, Jonas Bergh, Thomas Hatschek, Theodoros Foukakis

**Supplementary Table S1.** The list of features used in the neural network-based classification

Smoothed: 25 µm: Nucleus: Area

Smoothed: 25 µm: Nucleus: Perimeter

Smoothed: 25 µm: Nucleus: Circularity

Smoothed: 25 µm: Nucleus: Max caliper

Smoothed: 25 µm: Nucleus: Min caliper

Smoothed: 25 µm: Nucleus: Eccentricity

Smoothed: 25 µm: Nucleus: Hematoxylin OD mean

Smoothed: 25 µm: Nucleus: Hematoxylin OD sum

Smoothed: 25 µm: Nucleus: Hematoxylin OD std dev

Smoothed: 25 µm: Nucleus: Hematoxylin OD max

Smoothed: 25 µm: Nucleus: Hematoxylin OD min

Smoothed: 25 µm: Nucleus: Hematoxylin OD range

Smoothed: 25 µm: Nucleus: DAB OD mean

Smoothed: 25 µm: Nucleus: DAB OD sum

Smoothed: 25 µm: Nucleus: DAB OD std dev

Smoothed: 25 µm: Nucleus: DAB OD max

Smoothed: 25 µm: Nucleus: DAB OD min

Smoothed: 25 µm: Nucleus: DAB OD range

Smoothed: 25 µm: Cell: Area

Smoothed: 25 µm: Cell: Perimeter

Smoothed: 25 µm: Cell: Circularity

Smoothed: 25 µm: Cell: Max caliper

Smoothed: 25 µm: Cell: Min caliper

Smoothed: 25 µm: Cell: Eccentricity

Smoothed: 25 µm: Cell: DAB OD mean

Smoothed: 25 µm: Cell: DAB OD std dev

Smoothed: 25 µm: Cell: DAB OD max

Smoothed: 25 µm: Cell: DAB OD min

Smoothed: 25 µm: Cytoplasm: DAB OD mean

Smoothed: 25 µm: Cytoplasm: DAB OD std dev

Smoothed: 25 µm: Cytoplasm: DAB OD max

Smoothed: 25 µm: Cytoplasm: DAB OD min

Smoothed: 25 µm: Nucleus/Cell area ratio

Smoothed: 25 µm: Nearby detection counts

Smoothed: 50 µm: Nucleus: Area

Smoothed: 50 µm: Nucleus: Perimeter

Smoothed: 50 µm: Nucleus: Circularity

Smoothed: 50 µm: Nucleus: Max caliper

Smoothed: 50 µm: Nucleus: Min caliper

Smoothed: 50 µm: Nucleus: Eccentricity

Smoothed: 50 µm: Nucleus: Hematoxylin OD mean

Smoothed: 50 µm: Nucleus: Hematoxylin OD sum

Smoothed: 50 µm: Nucleus: Hematoxylin OD std dev

Smoothed: 50 µm: Nucleus: Hematoxylin OD max

Smoothed: 50 µm: Nucleus: Hematoxylin OD min

Smoothed: 50 µm: Nucleus: Hematoxylin OD range

Smoothed: 50 µm: Nucleus: DAB OD mean

Smoothed: 50 µm: Nucleus: DAB OD sum

Smoothed: 50 µm: Nucleus: DAB OD std dev

Smoothed: 50 µm: Nucleus: DAB OD max

Smoothed: 50 µm: Nucleus: DAB OD min

Smoothed: 50 µm: Nucleus: DAB OD range

Smoothed: 50 µm: Cell: Area

Smoothed: 50 µm: Cell: Perimeter

Smoothed: 50 µm: Cell: Circularity

Smoothed: 50 µm: Cell: Max caliper

Smoothed: 50 µm: Cell: Min caliper

Smoothed: 50 µm: Cell: Eccentricity

Smoothed: 50 µm: Cell: DAB OD mean

Smoothed: 50 µm: Cell: DAB OD std dev

Smoothed: 50 µm: Cell: DAB OD max

Smoothed: 50 µm: Cell: DAB OD min

Smoothed: 50 µm: Cytoplasm: DAB OD mean

Smoothed: 50 µm: Cytoplasm: DAB OD std dev

Smoothed: 50 µm: Cytoplasm: DAB OD max

Smoothed: 50 µm: Cytoplasm: DAB OD min

Smoothed: 50 µm: Nucleus/Cell area ratio

Smoothed: 50 µm: Nearby detection counts

Nucleus: Area

Nucleus: Perimeter

Nucleus: Circularity

Nucleus: Max caliper

Nucleus: Min caliper

Nucleus: Eccentricity

Nucleus: Hematoxylin OD mean

Nucleus: Hematoxylin OD sum

Nucleus: Hematoxylin OD std dev

Nucleus: Hematoxylin OD max

Nucleus: Hematoxylin OD min

Nucleus: Hematoxylin OD range

Nucleus: DAB OD mean

Nucleus: DAB OD sum

Nucleus: DAB OD std dev

Nucleus: DAB OD max

Nucleus: DAB OD min

Nucleus: DAB OD range

Cell: Area

Cell: Perimeter

Cell: Circularity

Cell: Max caliper

Cell: Min caliper

Cell: Eccentricity

Cell: DAB OD mean

Cell: DAB OD std dev

Cell: DAB OD max

Cell: DAB OD min

Cytoplasm: DAB OD mean

Cytoplasm: DAB OD std dev

Cytoplasm: DAB OD max

Cytoplasm: DAB OD min

Nucleus/Cell area ratio

**Supplementary Table S2.** Concordant and discordant gene-sets with Ki67 expression levels and TK1 activity. FDR: false discovery rate; NES: normalized enrichment score; pval: p value

Concordant gene-sets

| **name** | **nes_tk1** | **pval_tk1** | **fdr_tk1** | **nes_ki67** | **pval_ki67** | **fdr_ki67** |
| --- | --- | --- | --- | --- | --- | --- |
| REACTOME_MITOTIC_M_M_G1_PHASES | 2.377 | 0.000 | 0.000 | 2.557 | 0.000 | 0.000 |
| REACTOME_DNA_REPLICATION | 2.274 | 0.000 | 0.000 | 2.574 | 0.000 | 0.000 |
| REACTOME_CELL_CYCLE_MITOTIC | 2.261 | 0.000 | 0.000 | 2.429 | 0.000 | 0.000 |
| REACTOME_MITOTIC_PROMETAPHASE | 2.260 | 0.000 | 0.000 | 2.311 | 0.000 | 0.000 |
| REACTOME_CELL_CYCLE | 2.190 | 0.000 | 0.001 | 2.372 | 0.000 | 0.000 |
| REACTOME_NEP_NS2_INTERACTS_WITH_THE_CELLULAR_EXPOR | 2.092 | 0.002 | 0.001 | 2.151 | 0.000 | 0.000 |
| REACTOME_M_G1_TRANSITION | 2.085 | 0.000 | 0.001 | 2.251 | 0.000 | 0.000 |
| REACTOME_SYNTHESIS_AND_INTERCONVERSION_OF_NUCLEOTI | 2.034 | 0.000 | 0.003 | 1.540 | 0.024 | 0.051 |
| REACTOME_ACTIVATION_OF_ATR_IN_RESPONSE_TO_REPLICAT | 2.025 | 0.000 | 0.003 | 1.833 | 0.004 | 0.005 |
| REACTOME_CELL_CYCLE_CHECKPOINTS | 2.023 | 0.000 | 0.003 | 2.506 | 0.000 | 0.000 |
| REACTOME_ACTIVATION_OF_THE_PRE_REPLICATIVE_COMPLEX | 2.021 | 0.000 | 0.003 | 1.719 | 0.005 | 0.014 |
| REACTOME_G1_S_TRANSITION | 2.014 | 0.000 | 0.003 | 2.336 | 0.000 | 0.000 |
| REACTOME_TRANSPORT_OF_MATURE_MRNA_DERIVED_FROM_AN_ | 2.006 | 0.000 | 0.003 | 2.174 | 0.000 | 0.000 |
| REACTOME_REGULATION_OF_ORNITHINE_DECARBOXYLASE_ODC | 1.980 | 0.000 | 0.005 | 2.243 | 0.000 | 0.000 |
| REACTOME_ASSEMBLY_OF_THE_PRE_REPLICATIVE_COMPLEX | 1.958 | 0.000 | 0.006 | 2.201 | 0.000 | 0.000 |
| REACTOME_G2_M_CHECKPOINTS | 1.950 | 0.000 | 0.006 | 1.940 | 0.000 | 0.002 |
| REACTOME_KINESINS | 1.948 | 0.000 | 0.006 | 2.133 | 0.000 | 0.000 |
| REACTOME_CYCLIN_A_B1_ASSOCIATED_EVENTS_DURING_G2_M | 1.934 | 0.000 | 0.007 | 2.037 | 0.000 | 0.000 |
| REACTOME_LATE_PHASE_OF_HIV_LIFE_CYCLE | 1.933 | 0.000 | 0.007 | 2.097 | 0.000 | 0.000 |
| REACTOME_TRANSPORT_OF_MATURE_TRANSCRIPT_TO_CYTOPLA | 1.920 | 0.000 | 0.007 | 2.116 | 0.000 | 0.000 |
| REACTOME_E2F_MEDIATED_REGULATION_OF_DNA_REPLICATIO | 1.897 | 0.000 | 0.009 | 1.899 | 0.000 | 0.003 |
| REACTOME_TRANSPORT_OF_RIBONUCLEOPROTEINS_INTO_THE_ | 1.885 | 0.002 | 0.010 | 1.984 | 0.002 | 0.001 |
| REACTOME_ANTIGEN_PROCESSING_UBIQUITINATION_PROTEAS | 1.868 | 0.000 | 0.011 | 1.787 | 0.000 | 0.008 |
| REACTOME_REGULATION_OF_MITOTIC_CELL_CYCLE | 1.866 | 0.002 | 0.011 | 2.633 | 0.000 | 0.000 |
| REACTOME_HIV_LIFE_CYCLE | 1.808 | 0.000 | 0.020 | 2.127 | 0.000 | 0.000 |
| REACTOME_APC_C_CDC20_MEDIATED_DEGRADATION_OF_MITOT | 1.808 | 0.002 | 0.019 | 2.620 | 0.000 | 0.000 |
| REACTOME_APC_C_CDH1_MEDIATED_DEGRADATION_OF_CDC20_ | 1.804 | 0.002 | 0.019 | 2.515 | 0.000 | 0.000 |
| REACTOME_ORC1_REMOVAL_FROM_CHROMATIN | 1.803 | 0.000 | 0.019 | 2.200 | 0.000 | 0.000 |
| REACTOME_FORMATION_OF_RNA_POL_II_ELONGATION_COMPLE | 1.803 | 0.002 | 0.018 | 1.872 | 0.000 | 0.004 |
| REACTOME_REGULATION_OF_GLUCOKINASE_BY_GLUCOKINASE_ | 1.802 | 0.000 | 0.018 | 2.007 | 0.000 | 0.001 |
| REACTOME_AUTODEGRADATION_OF_CDH1_BY_CDH1_APC_C | 1.801 | 0.000 | 0.018 | 2.554 | 0.000 | 0.000 |
| REACTOME_CDT1_ASSOCIATION_WITH_THE_CDC6_ORC_ORIGIN | 1.794 | 0.002 | 0.019 | 2.141 | 0.000 | 0.000 |
| REACTOME_HOST_INTERACTIONS_OF_HIV_FACTORS | 1.782 | 0.000 | 0.020 | 2.489 | 0.000 | 0.000 |
| REACTOME_HIV_INFECTION | 1.766 | 0.000 | 0.023 | 2.338 | 0.000 | 0.000 |
| REACTOME_INTERACTIONS_OF_VPR_WITH_HOST_CELLULAR_PR | 1.765 | 0.004 | 0.022 | 2.135 | 0.000 | 0.000 |
| REACTOME_P53_INDEPENDENT_G1_S_DNA_DAMAGE_CHECKPOIN | 1.750 | 0.004 | 0.025 | 2.321 | 0.000 | 0.000 |
| REACTOME_REGULATORY_RNA_PATHWAYS | 1.747 | 0.008 | 0.025 | 1.767 | 0.007 | 0.010 |
| REACTOME_CDK_MEDIATED_PHOSPHORYLATION_AND_REMOVAL_ | 1.744 | 0.006 | 0.025 | 2.213 | 0.000 | 0.000 |
| REACTOME_S_PHASE | 1.743 | 0.002 | 0.025 | 2.328 | 0.000 | 0.000 |
| REACTOME_MITOTIC_G1_G1_S_PHASES | 1.743 | 0.000 | 0.024 | 2.277 | 0.000 | 0.000 |
| REACTOME_SYNTHESIS_OF_DNA | 1.741 | 0.000 | 0.024 | 2.361 | 0.000 | 0.000 |
| REACTOME_MICRORNA_MIRNA_BIOGENESIS | 1.740 | 0.008 | 0.024 | 1.894 | 0.006 | 0.003 |
| REACTOME_ER_PHAGOSOME_PATHWAY | 1.730 | 0.002 | 0.026 | 1.860 | 0.000 | 0.004 |
| REACTOME_TRNA_AMINOACYLATION | 1.719 | 0.000 | 0.028 | 2.362 | 0.000 | 0.000 |
| REACTOME_SCF_BETA_TRCP_MEDIATED_DEGRADATION_OF_EMI | 1.715 | 0.002 | 0.028 | 2.262 | 0.000 | 0.000 |
| REACTOME_CHROMOSOME_MAINTENANCE | 1.712 | 0.000 | 0.028 | 2.050 | 0.000 | 0.000 |
| REACTOME_CYCLIN_E_ASSOCIATED_EVENTS_DURING_G1_S_TR | 1.709 | 0.002 | 0.029 | 2.266 | 0.000 | 0.000 |
| REACTOME_RNA_POL_II_PRE_TRANSCRIPTION_EVENTS | 1.708 | 0.007 | 0.029 | 1.903 | 0.000 | 0.003 |
| REACTOME_DEPOSITION_OF_NEW_CENPA_CONTAINING_NUCLEO | 1.704 | 0.004 | 0.029 | 2.090 | 0.000 | 0.000 |
| REACTOME_MITOCHONDRIAL_TRNA_AMINOACYLATION | 1.679 | 0.006 | 0.034 | 2.133 | 0.000 | 0.000 |
| REACTOME_VIF_MEDIATED_DEGRADATION_OF_APOBEC3G | 1.674 | 0.006 | 0.035 | 2.209 | 0.000 | 0.000 |
| REACTOME_METABOLISM_OF_NON_CODING_RNA | 1.672 | 0.005 | 0.035 | 2.229 | 0.000 | 0.000 |
| REACTOME_AUTODEGRADATION_OF_THE_E3_UBIQUITIN_LIGAS | 1.665 | 0.002 | 0.037 | 2.203 | 0.000 | 0.000 |
| REACTOME_G1_S_SPECIFIC_TRANSCRIPTION | 1.653 | 0.022 | 0.040 | 1.886 | 0.004 | 0.003 |
| REACTOME_TCA_CYCLE_AND_RESPIRATORY_ELECTRON_TRANSP | 1.627 | 0.000 | 0.047 | 2.311 | 0.000 | 0.000 |
| REACTOME_MRNA_PROCESSING | 1.624 | 0.002 | 0.047 | 2.191 | 0.000 | 0.000 |
| REACTOME_CROSS_PRESENTATION_OF_SOLUBLE_EXOGENOUS_A | 1.624 | 0.009 | 0.046 | 2.124 | 0.000 | 0.000 |
| REACTOME_DESTABILIZATION_OF_MRNA_BY_AUF1_HNRNP_D0 | 1.622 | 0.011 | 0.046 | 2.298 | 0.000 | 0.000 |
| REACTOME_RESPIRATORY_ELECTRON_TRANSPORT_ATP_SYNTHE | 1.589 | 0.008 | 0.058 | 2.341 | 0.000 | 0.000 |
| REACTOME_SIGNALING_BY_WNT | 1.583 | 0.004 | 0.060 | 2.045 | 0.000 | 0.000 |
| REACTOME_P53_DEPENDENT_G1_DNA_DAMAGE_RESPONSE | 1.579 | 0.009 | 0.061 | 2.123 | 0.000 | 0.000 |
| REACTOME_PROCESSING_OF_CAPPED_INTRON_CONTAINING_PR | 1.562 | 0.002 | 0.068 | 2.189 | 0.000 | 0.000 |
| REACTOME_RESPIRATORY_ELECTRON_TRANSPORT | 1.533 | 0.013 | 0.082 | 2.271 | 0.000 | 0.000 |
| REACTOME_ACTIVATION_OF_NF_KAPPAB_IN_B_CELLS | 1.532 | 0.020 | 0.082 | 2.045 | 0.000 | 0.000 |
| REACTOME_INHIBITION_OF_THE_PROTEOLYTIC_ACTIVITY_OF | 1.521 | 0.043 | 0.087 | 2.133 | 0.000 | 0.000 |
| REACTOME_G0_AND_EARLY_G1 | 1.519 | 0.033 | 0.087 | 1.878 | 0.002 | 0.004 |
| REACTOME_SCFSKP2_MEDIATED_DEGRADATION_OF_P27_P21 | 1.517 | 0.023 | 0.087 | 2.179 | 0.000 | 0.000 |

Discordant gene-sets

| **name** | **nes_tk1** | **pval_tk1** | **fdr_tk1** | **nes_ki67** | **pval_ki67** | **fdr_ki67** |
| --- | --- | --- | --- | --- | --- | --- |
| REACTOME_INFLUENZA_VIRAL_RNA_TRANSCRIPTION_AND_REP | -2.860 | 0 | 0.000 | 1.791 | 0.000 | 0.008 |
| REACTOME_3_UTR_MEDIATED_TRANSLATIONAL_REGULATION | -2.777 | 0 | 0.000 | 1.785 | 0.000 | 0.008 |
| REACTOME_SRP_DEPENDENT_COTRANSLATIONAL_PROTEIN_TAR | -2.691 | 0 | 0.000 | 1.611 | 0.002 | 0.032 |
| REACTOME_NONSENSE_MEDIATED_DECAY_ENHANCED_BY_THE_E | -2.651 | 0 | 0.000 | 1.673 | 0.000 | 0.022 |
| REACTOME_TRANSLATION | -2.406 | 0 | 0.000 | 1.829 | 0.000 | 0.005 |
| REACTOME_INFLUENZA_LIFE_CYCLE | -2.277 | 0 | 0.000 | 2.100 | 0.000 | 0.000 |
| REACTOME_FORMATION_OF_THE_TERNARY_COMPLEX_AND_SUBS | -2.178 | 0 | 0.001 | 1.845 | 0.000 | 0.005 |
| REACTOME_ACTIVATION_OF_THE_MRNA_UPON_BINDING_OF_TH | -2.157 | 0 | 0.001 | 1.744 | 0.002 | 0.012 |
| REACTOME_METABOLISM_OF_MRNA | -1.738 | 0 | 0.053 | 2.039 | 0.000 | 0.000 |
| REACTOME_METABOLISM_OF_RNA | -1.526 | 0 | 0.124 | 2.248 | 0.000 | 0.000 |

**Supplementary Table S3.** Sensitivity analyses on the association between TK1 increase from baseline to cycle 2 and event free and overall survival

| **TK1 increase from baseline to cycle2^a^** | **Cox proportional hazards regression**  **Hazard ratio (95% confidence interval)** | | | |
| --- | --- | --- | --- | --- |
|  | **Adjusted Model ^b^** | | **Full-Model ^c^** | |
|  | **HR (95% CI)** | **P** | **HR (95% CI)** | **P** |
| **Event Free Survival** | | | | |
| Low | 1.00 (reference) |  | 1.00 (reference) |  |
| High | 0.51 (0.26, 1.00) | 0.05 | 0.54 [0.27, 1.06] | 0.07 |
| **Overall Survival** | | | | |
| Low | 1.00 (reference) |  | 1.00 (reference) |  |
| High | 0.48 (0.23, 1.00) | 0.05 | 0.49 [0.23, 1.03] | 0.06 |

^a^ Low/high delineation of TK1 increase from baseline to cycle2 was determined according to their median value.

^b^ Stratified by ER status (negative, positive) due to PH assumption violation (P=0.04) and adjusted for tumor size (≥50 mm, <50 mm), regional lymph node (negative, positive) and Ki67 (<12%, ≥12%).

^c^ Stratified by ER status (negative, positive) due to PH assumption violation (P=0.04) and adjusted for tumor size (≥50 mm, <50 mm), age at diagnosis (continuous), regional lymph node (negative, positive), Ki67 (<12%, ≥12%).

Abbreviations: HR, hazard ratio; CI, confidence interval; ER, estrogen receptor; PH, proportional hazards.

**Supplementary table S4.** Multivariable analysis for TK1 activity (baseline and change to post-cycle 2) with event-free survival as clinical endpoint when adding pathologic complete response to the model

| **TK1** | **Cox regression**  **Hazard Ratio (95% confidence interval)** | |
| --- | --- | --- |
|  | **Adjusted Model ^b^** | |
|  | **HR (95% CI)** | **P** |
| **TK1 at baseline ^a^** |  |  |
| High vs low (all) | 1.59 [0.83, 3.06] | 0.17 |
| High vs low (HR+) | 1.82 [0.78, 4.23] | 0.17 |
| High vs low (TN) | 0.89 [0.30, 2.66] | 0.84 |
| **TK1 increase from baseline to cycle2 ^b^** |  |  |
| High vs low (all) | **0.50 [0.26, 0.97]** | **0.04** |
| High vs low (HR+) | 0.52 [0.23, 1.19] | 0.12 |
| High vs low (TN) | 0.52 [0.15, 1.83] | 0.31 |

^a^ Stratified by ER status (negative, positive) due to PH assumption violation (P=0.06) and adjusted for pCR status and regional lymph node (negative, positive).

^b^ Low/high delineations of TK/TK kinetics were determined according to their median value.

^c^ Stratified by ER status (negative, positive) due to PH assumption violation (P=0.03) and adjusted for pCR status, tumor size (>50mm, <50mm), and regional lymph node (negative, positive).

**Supplementary Table S5.** Univariate and multivariable analyses of TK1 per 10-fold increase between baseline and cycle 2, for event-free and overall survival

| **TK1 increase from baseline to cycle2** | **Cox proportional hazards regression**  **Hazard ratio (95% confidence interval) for event-free survival** | | | | **Cox proportional hazards regression**  **Hazard ratio (95% confidence interval) for overall survival** | | | |
| --- | --- | --- | --- | --- | --- | --- | --- | --- |
|  | **Unadjusted Model** | | **Adjusted Model ^a^** | | **Unadjusted Model** | | **Adjusted Model ^a^** | |
|  | **HR (95% CI)** | **P** | **HR (95% CI)** | **P** | **HR (95% CI)** | **P** | **HR (95% CI)** | **P** |
|  |  |  |  |  |  |  |  |  |
| Per 10-fold increase | 0.69 (0.54, 0.88) | 0.002 | 0.81 (0.62, 1.05) | 0.10 | 0.66 (0.51, 0.86) | 0.002 | 0.78 (0.60, 1.03) | 0.08 |

^a^ Stratified by ER status (negative, positive) due to PH assumption violation (P=0.04) and adjusted for tumor size (≥50 mm, <50 mm), regional lymph node (negative, positive).

Abbreviations: EFS, event-free survival; OS, overall survival; HR, hazard ratio; CI, confidence interval; ER, estrogen receptor; PH, proportional hazards

**Supplementary Figure S1:** Flowchart of data availability in the PROMIX study cohort

**
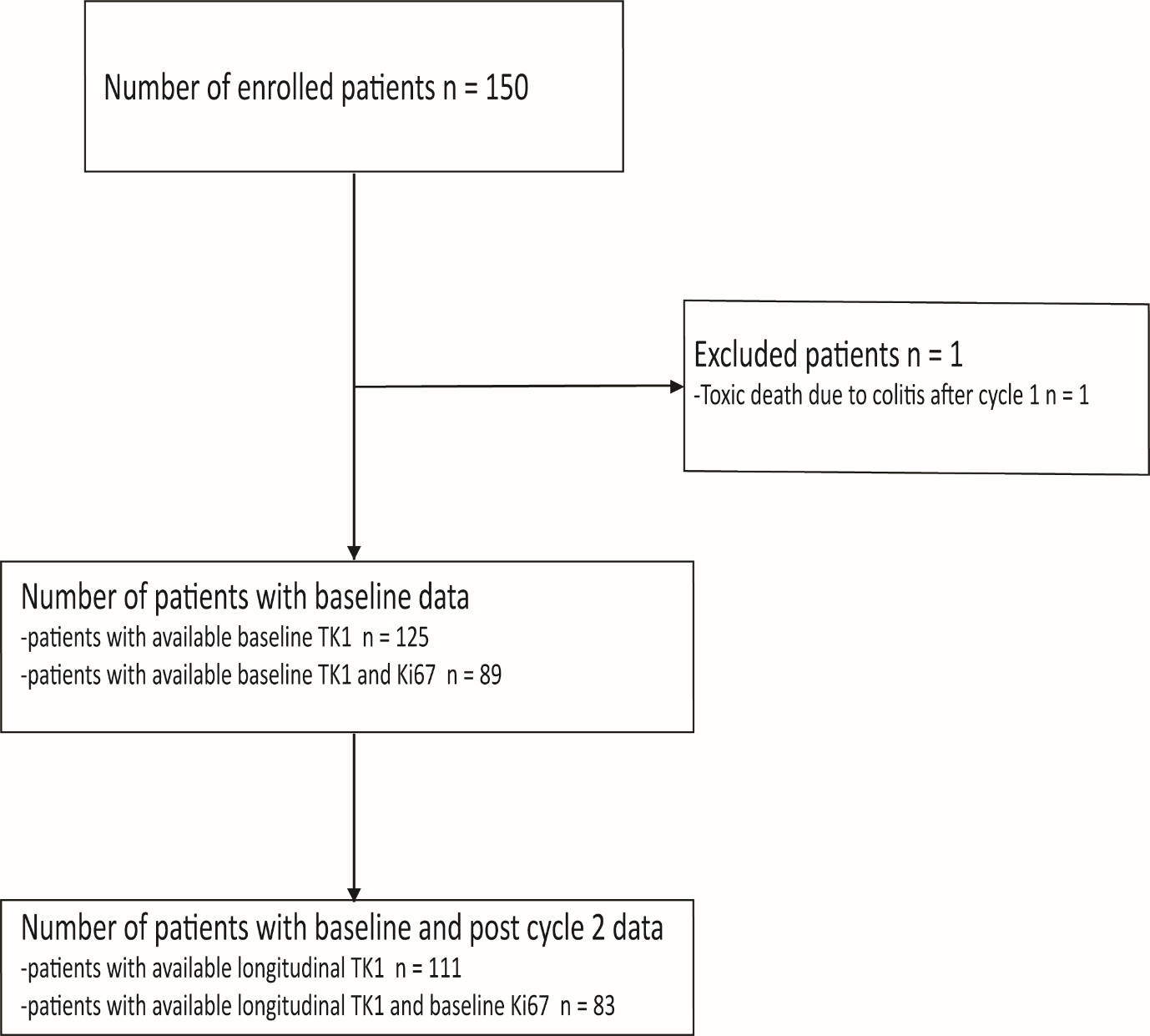
**

**Supplementary Figure S2.** Kaplan-Meier curves of event-free survival (A) and overall survival (B) according to level of TK1 increase from baseline to post-cycle 2, using the median increase as cut-off.


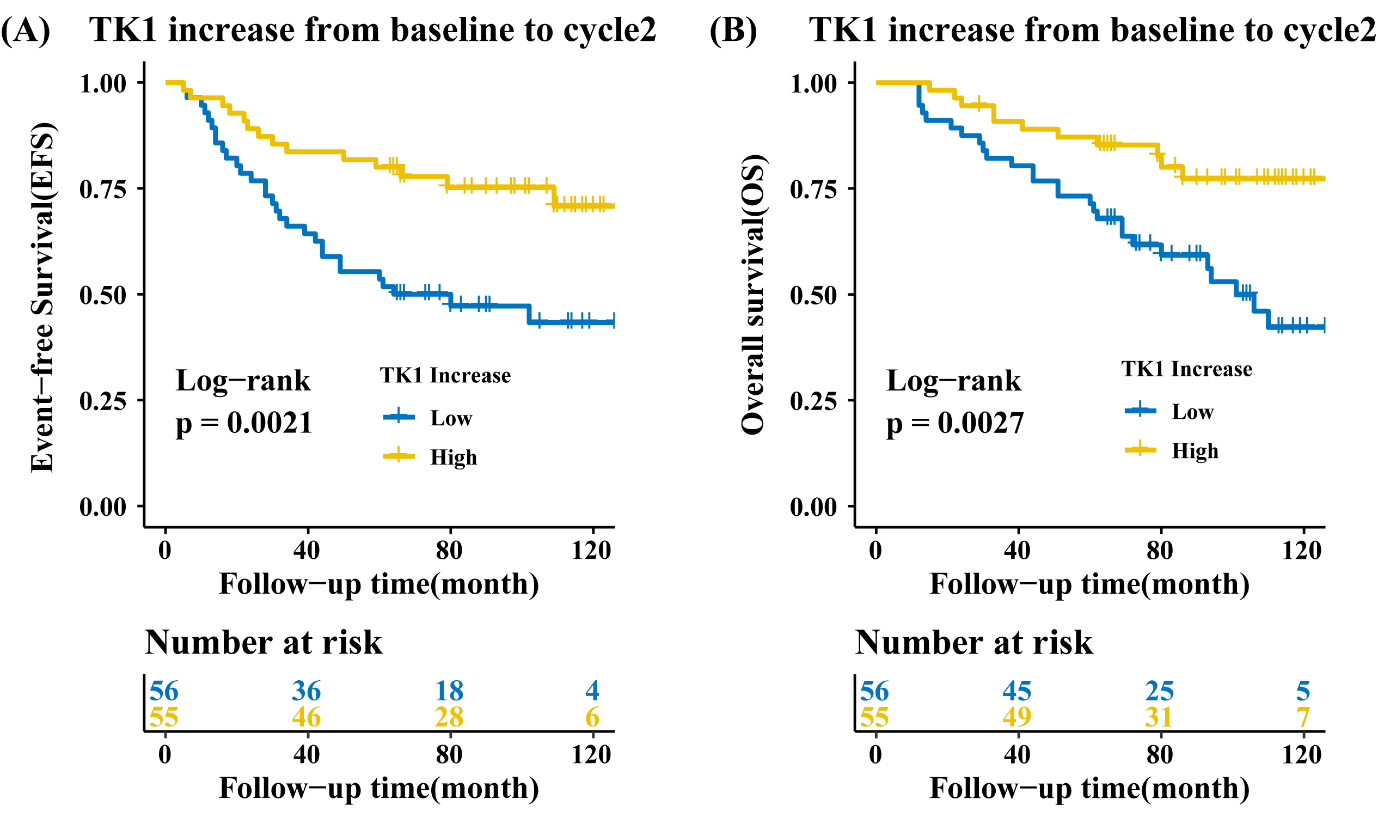


**REMARK checklist**

| **Item to be reported** | | **Page no.** |
| --- | --- | --- |
| **INTRODUCTION** | |  |
| 1 | State the marker examined, the study objectives, and any pre-specified hypotheses. | 5 |
| **MATERIALS AND METHODS** | |  |
| *Patients* | |  |
| 2 | Describe the characteristics (e.g., disease stage or co-morbidities) of the study patients, including their source and inclusion and exclusion criteria. | 6 |
| 3 | Describe treatments received and how chosen (e.g., randomized or rule-based). |  |
| *Specimen characteristics* | |  |
| 4 | Describe type of biological material used (including control samples) and methods of preservation and storage. | 7,9,10 |
| *Assay methods* | |  |
| 5 | Specify the assay method used and provide (or reference) a detailed protocol, including specific reagents or kits used, quality control procedures, reproducibility assessments, quantitation methods, and scoring and reporting protocols. Specify whether and how assays were performed blinded to the study endpoint. | 7-10 |
| *Study design* | |  |
| 6 | State the method of case selection, including whether prospective or retrospective and whether stratification or matching (e.g., by stage of disease or age) was used. Specify the time period from which cases were taken, the end of the follow-up period, and the median follow-up time. | 6 |
| 7 | Precisely define all clinical endpoints examined. | 6 |
| 8 | List all candidate variables initially examined or considered for inclusion in models. | 10-12 |
| 9 | Give rationale for sample size; if the study was designed to detect a specified effect size, give the target power and effect size. | 6 |
| *Statistical analysis methods* | |  |
| 10 | Specify all statistical methods, including details of any variable selection procedures and other model-building issues, how model assumptions were verified, and how missing data were handled. | 10-12 |
| 11 | Clarify how marker values were handled in the analyses; if relevant, describe methods used for cutpoint determination. | 10-12 |
| **RESULTS** | |  |
| *Data* | |  |
| 12 | Describe the flow of patients through the study, including the number of patients included in each stage of the analysis (a diagram may be helpful) and reasons for dropout. Specifically, both overall and for each subgroup extensively examined report the numbers of patients and the number of events. | Figure 1 |
| 13 | Report distributions of basic demographic characteristics (at least age and sex), standard (disease-specific) prognostic variables, and tumor marker, including numbers of missing values. | Table 1 |
| *Analysis and presentation* | |  |
| 14 | Show the relation of the marker to standard prognostic variables. | 13 |
| 15 | Present univariable analyses showing the relation between the marker and outcome, with the estimated effect (e.g., hazard ratio and survival probability). Preferably provide similar analyses for all other variables being analyzed. For the effect of a tumor marker on a time-to-event outcome, a Kaplan-Meier plot is recommended. | Table 2,3 |
| 16 | For key multivariable analyses, report estimated effects (e.g., hazard ratio) with confidence intervals for the marker and, at least for the final model, all other variables in the model. | Table 2,3 |
| 17 | Among reported results, provide estimated effects with confidence intervals from an analysis in which the marker and standard prognostic variables are included, regardless of their statistical significance. | Table 2,3, supplement |
| 18 | If done, report results of further investigations, such as checking assumptions, sensitivity analyses, and internal validation. | Supp table S3 |
| **DISCUSSION** | |  |
| 19 | Interpret the results in the context of the pre-specified hypotheses and other relevant studies; include a discussion of limitations of the study. | 16-18 |
| 20 | Discuss implications for future research and clinical value. | 16-18 |
